# Albuminous Conditions of the Blood, Etc.

**Published:** 1877-08-20

**Authors:** 


DR. J. L. HAMILTON, of Georgia, informs us that he has found no remedy so useful in albuminous conditions of the blood and dropsical accumulations as the following:

R. Acetate squills............ ..oz.ij.
Acetate pot.
Spts. nitre dulc ..........aa oz.j.
Tinct. digitalis....... ......dr.i M.
Teaspoonful every four to six hours.

---

FLUID EXTRACT OF KIDNEY LEAF COM.—The above remedy is highly extolled in that opprobrium of medicine—Bright's disease of the kidneys.

